# Sind wir auf einen Bündnisfall vorbereitet? Was können wir aus der Versorgung ukrainischer Kriegsverletzter in den vergangenen 3½ Jahren lernen?

**DOI:** 10.1007/s00113-026-01680-4

**Published:** 2026-01-20

**Authors:** Michael J. Raschke, Alex Michael Lechleuthner, Tobias Hirsch

**Affiliations:** 1 Klinik für Unfall‑, Hand- und Wiederherstellungschirurgie, Universitätsklinikum Münster, Waldeyerstr. 1, 48149 Münster, Deutschland; 2 Rettungsdienst der Stadt Köln, SPoC.NRW, Köln, Deutschland; 3 Klinik für Plastische Chirurgie, Universitätsklinikum Münster, Münster, Deutschland

**Keywords:** Kriegsverletzungen, Extremitätentrauma, Interdisziplinarität, Patientenzuweisung, TraumaNetzwerk DGU, War injuries, Extremity trauma, Interdisciplinarity, Patient distribution, DGU Trauma Network

## Abstract

Seit Beginn des russischen Invasionskrieges wurden über 2000 Kriegsverletzte im westlichen Europa behandelt. Über das Kleeblattsystem vom SpoC.NRW wurden 324 Patienten verteilt. Ermittelt wurden die Verletzungsformen und die erforderlichen Fachdisziplinen sowie deren Verteilung, übertragen auf Strukturen der Deutschen Gesellschaft für Unfallchirurgie (lokales, regionales und überregionales Traumazentrum). Erwartungsgemäß waren 94 % der Verletzten zwischen 21 und 60 Jahre alt. 66 % waren Extremitätenverletzungen. Hiervon 26,6 % an der oberen- und 39,8 % an der unteren Extremität. Ausgewertet wurden die benötigten Fachabteilungen in Abhängigkeit von den betroffenen Lokalisationen. Hier zeigte sich, dass im überwiegenden Maß neben unfallchirurgischer (89 %) vermehrt plastisch-chirurgische (61 %) und allgemeinchirurgische Kompetenz (8 %) beansprucht wurden. Bei interdisziplinären Behandlungen waren die typischen Kopffächer (NCH, HNO, Augenheilkunde, MKG) in 19 % involviert. Ukrainische Kriegsverletzte, die in den Krankenhäusern in NRW, welche bereit waren, diese Patienten aufzunehmen, konnten bisher sehr gut versorgt werden. Die Verteilung der in NRW behandelten ukrainischen Kriegsverletzten zeigt eine Konzentration auf die überregionalen Traumazentren (55 %). Im „Bündnisfall“ wird mit über 1000 behandlungsbedürftigen Patientinnen und Patienten/Tag, die nach Deutschland kommen und hier weiterverteilt werden müssen, gerechnet. Die erstmals vorliegenden Daten geben Hinweise auf zukünftige Erfordernisse.

## Abstract (en)

Since the beginning of the Russian invasion, approximately 2000 war casualties have been treated in Western Europe and of these 324 patients were distributed to North Rhine-Westphalia (NRW) via the SPoC.NRW cloverleaf system. The types of injuries and the required medical specialties were determined as well as their distribution transferred to the structures of the German Society for Trauma Surgery (DGU local, regional, supraregional trauma centers). As expected, 94% of the injured were between 21 and 60 years old, 66% of the injuries affected the upper extremities (26.6%) and the lower extremities (39.8%). The required departments were evaluated based on the affected locations. This revealed that in addition to trauma surgery (89%), plastic surgery (61%) and general surgery (8%) were predominantly required. In interdisciplinary treatment the typical head and neck specialties (neurology, ENT, ophthalmology, maxillofacial surgery = 19%) were involved. Ukrainian war casualties treated in hospitals in NRW that were willing to accept such patients have so far received very good care. The distribution of Ukrainian war casualties treated in NRW shows a concentration in supraregional trauma centers (55%). In the event of a military intervention, it is anticipated that over 1000 patients requiring treatment will arrive in Germany every day. These first data provide insights into future needs of medical care.

Die Behandlung kriegsverletzter Patientinnen und Patienten stellt das Gesundheitssystem vor besondere Herausforderungen. Diese Patienten weisen meist komplexe Verletzungen auf, gelangen erst nach Wochen und Monaten in unsere Behandlung, die meisten Wunden sind mit sog. Problemkeimen (z. B. 4-fach Multi Resistente Gram Negative Erreger [4 MRGN]) kontaminiert. Zusätzlich besteht meist eine erhebliche Sprach- und Informationsbarriere.

Seit Beginn des russischen Invasionskrieges auf die Ukraine am 24.02.2022 wurden mehr als 2000 überwiegend kriegsverletzte Patientinnen und Patienten im westlichen Europa behandelt. Hiervon entfallen über 1500 Patienten, die auf unterschiedlichen Wegen hergekommen sind, allein auf die Bundesrepublik Deutschland [1].

Die Bundeswehr und das Bundesamt für Bevölkerungsschutz und Katastrophenhilfe (BBK) rechnen für einen etwaigen „Bündnisfall“ mit über 1000 behandlungsbedürftigen Verletzten/Tag, die nach Deutschland kommen, hier versorgt und weiterverteilt werden müssen [2].

In diesem Fall wären wir mit einer erheblich höheren Zahl an Verletzten mit einem deutlich akuteren Verletzungsmuster konfrontiert.

Was können wir von den vergangenen dreieinhalb Jahren der Versorgung ukrainischer Kriegsverletzter für die Zukunft lernen? Sind wir ausreichend auf ein derartiges Szenario vorbereitet?

Von den ukrainischen Verletzten, die über das Kleeblattsystem nach NRW kamen und vom SPoC.NRW verteilt wurden, sind 324 untersucht worden. Die Unterlagen mit den beschriebenen Verletzungen lagen dabei in nichtstandardisierter Form vor. Die Aufbereitung der Patientenunterlagen für diese Untersuchung wurde im Sinne der DSGVO mit dem Datenschutzbeauftragten des Universitätsklinikum Münster abgestimmt sowie von der Ethik-Kommission Westfalen-Lippe (AZ: 2025-492-f‑S, 07.07.2025) genehmigt. Dies entspricht 22 % aller in der BRD versorgten ukrainischer Patienten.

Die vorliegende Auswertung beschreibt erstmals die Schwere der Verletzungen und deren Verteilung auf die an der Traumaversorgung beteiligten Kliniken, größtenteils in den Traumanetzwerken der DGU (Deutschen Gesellschaft für Unfallchirurgie) organisiert.

Ziel dieser retrospektiven Analyse ist es, die Verletzungsformen zu analysieren und daraus die für die Behandlung erforderlichen Fachdisziplinen zu ermitteln, deren Verteilung auf die Krankenhäuser darzustellen, sowie diese Daten exemplarisch auf die bewährten Strukturen der DGU Traumanetzwerke (lokales, regionales und überregionales Traumazentrum) zu spiegeln.

## Material und Methoden

Die zur Verfügung gestellten Unterlagen enthielten weder Geschlecht, Namen, Geburtsdaten noch sonstige personenbezogene Identifikatoren. Das Alter selbst war in Alterskohorten (1–10, 11–20, 21–40, 41–60, > 60) kodiert.

Aus diesen Unterlagen wurden die Verletzungen nach Art und Körperregion klassifiziert, sodass die Verletzungen nicht nur isoliert ausgewertet, sondern für jeden Patienten auch ein Verletzungsprofil in standardisierter Form erstellt und ausgewertet wurde. Die Daten wurden vom Ministerium für Arbeit, Gesundheit und Soziales – Nordrhein-Westfalen (MAGS) zur Verfügung gestellt. Auflage war die Einhaltung von strengen Datenschutzrichtlinien, die keine Nachverfolgung der behandelten Patienten möglich machen.

Die Zuordnung der aufbereiteten Verletztenprofile zu den einzelnen, erforderlichen Fachgebieten erfolgte in einem verblindeten Expertenpanel der Autoren. Ziel war eine standardisierte Zuordnung der anonymisierten Fälle zu verletzten Körperregion(en), Verletzungsart(en) und erforderlicher Fachdisziplin(en). Diese erfolgte auf Grundlage der dokumentierten ICD-10-Codes und des Patientenalters. Die Beurteilung erfolgte durch die drei Autoren (Facharzt für Unfallchirurgie, Facharzt für Plastische und Rekonstruktive Chirurgie, Facharzt für Chirurgie und ärztlichem Leiter des SPoC. NRW).

Zunächst wurden dabei die übereinstimmenden Zuordnungen abgeschlossen. Im zweiten Schritt wurden anschließend die primär nicht übereinstimmend eingeschätzten Fälle erneut bewertet und konsentiert. Letztlich bleibt allerdings bei allen Beurteilungen und Zuordnungen, für die nur die primären, medizinischen Unterlagen zur Verfügung standen, eine Restunsicherheit hinsichtlich der Eindeutigkeit der Zuordnung, da keine weiteren Daten zur klinischen Diagnostik und Therapie im Verlauf vorliegen.

## Ergebnisse

Im Folgenden werden die Ergebnisse der Auswertung der in NRW im Zeitraum von April 2022 bis Juli 2025 behandelten ukrainischen Kriegsverletzten dargestellt.

**Abb. 1:**
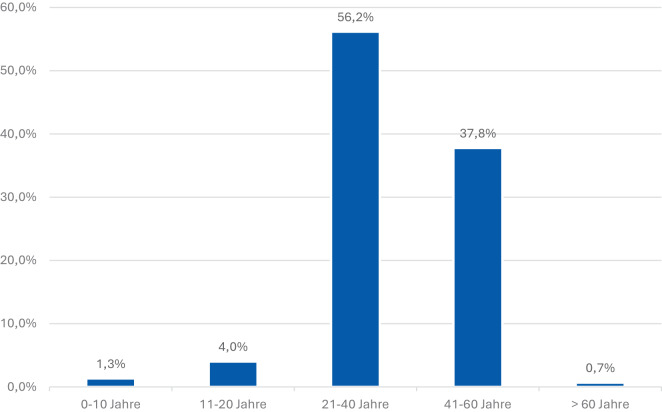
Alterskohorten ukrainischer Kriegsverletzter.. Altersverteilung der behandelten Patienten. Die beiden größten Gruppen entfallen auf die Alter von 21 bis 40 Jahren und von 41 bis 60 Jahren

Abb. 1 zeigt die Verteilung der behandelten ukrainischen Kriegsverletzten nach Alterskohorten. Die größte Gruppe entfällt mit 56,2 % auf das Altersspektrum von 21 bis 40 Jahren, gefolgt von 37,8 % im Alter von 41 bis 60 Jahren. Somit sind die meisten der Patienten im wehrfähigen Alter. Die Verletzungen sind weitestgehend bei Kriegshandlungen entstanden. Deutlich kleiner sind die Gruppen der jüngeren Patienten: Nur 4,0 % entfallen auf die Alterskohorte von 11 bis 20 Jahren und nur 1,3 % auf die Alterskohorte von 0 bis 10 Jahren. In der Alterskohorte über 60 Jahren waren nur 0,7 % der Verletzten enthalten.

**Abb. 2:**
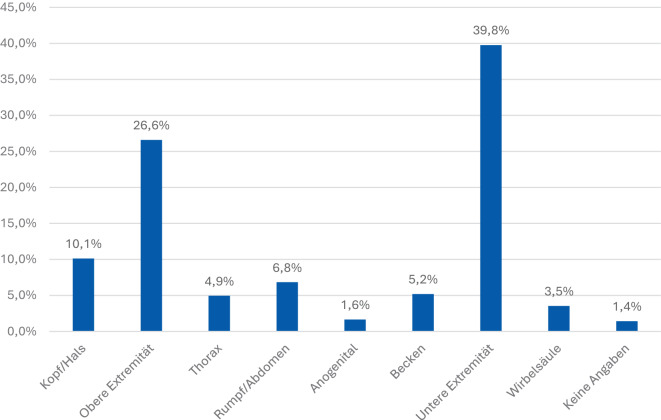
Lokalisation der Einzelverletzungen (Körperregionen). Häufigste betroffene Körperregionen waren die unteren Extremitäten, gefolgt von den oberen Extremitäten

Die Auswertung der verletzten Körperregionen zeigt ein deutliches Muster (Abb. 2): Am häufigsten betroffen sind die unteren Extremitäten mit 39,8 %, gefolgt von den oberen Extremitäten mit 26,6 %. Weitere Lokalisationen sind der Kopf‑/Halsbereich (10,1 %) sowie Rumpf/Abdomen (6,8 %). Verletzungen am Thorax (4,9 %), Becken (5,2 %) und an der Wirbelsäule (3,5 %). Diese sind seltener dokumentiert, während anogenitale Verletzungen mit 1,6 % lediglich eine kleine Gruppe darstellen. Bei 1,4 % lagen keine Angaben zur Lokalisation vor.

Das Verteilungsmuster zeigt die Dominanz von Extremitätenverletzungen und verdeutlicht den hohen Bedarf an traumatologisch-orthopädischer und rekonstruktiver Versorgung.

**Abb. 3:**
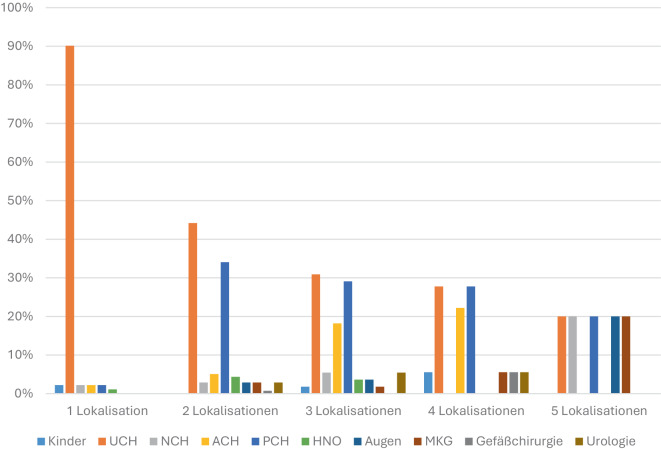
Anzahl der benötigten Fachabteilungen in Abhängigkeit von der Anzahl der betroffenen Körperregion. Bei Fällen mit einer Lokalisation entfiel der überwiegende Anteil auf die Unfallchirurgie (UCH)

Abb. 3 zeigt die Verteilung der beteiligten Fachabteilungen in Abhängigkeit von der Anzahl der betroffenen Körperregionen. Bei Fällen mit einer Lokalisation entfiel der überwiegende Anteil auf die Unfallchirurgie (UCH). Mit zunehmender Zahl der Lokalisationen verringerte sich der relative Anteil der UCH, während die Beteiligung weiterer Fachdisziplinen zunahm.

Bei zwei Körperregionen traten neben der UCH vermehrt plastisch-chirurgische (PCH) und allgemein-/viszeralchirurgische (ACH) Abteilungen hinzu. Ab drei Körperregionen zeigte sich ein deutlicher Anstieg interdisziplinärer Behandlungsverläufe unter Einbeziehung der Neurochirurgie (NCH), Hals-Nasen Ohren-Heilkunde (HNO), Augenheilkunde sowie Mund‑, Kiefer- und Gesichtschirurgie (MKG).

Mit vier oder mehr Körperregionen waren in der Regel mehrere chirurgische Fachrichtungen beteiligt, wobei Kombinationen aus UCH, PCH und ACH dominierten. Fälle mit fünf oder mehr Lokalisationen wiesen den höchsten Grad interdisziplinärer Beteiligung auf. Insgesamt zeigte sich ein deutlicher Zusammenhang zwischen der Zahl der betroffenen Lokalisationen und dem Umfang der interdisziplinären Versorgung.

**Abb. 4:**
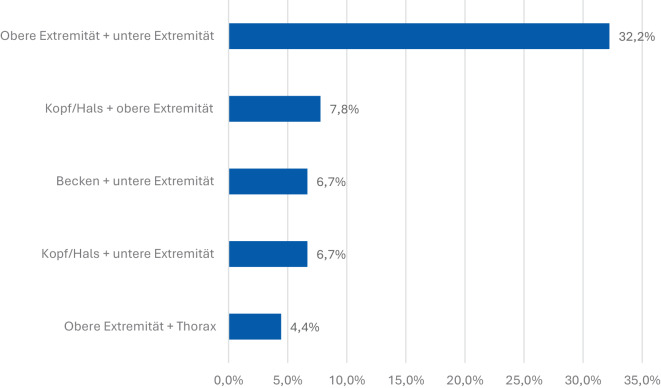
Die 5 häufigsten Kombinationen von verletzten Körperregionen (Verletzungsprofil). Am häufigsten waren obere und untere Extremitäten gemeinsam betroffen

Die Auswertung der häufigsten Kombinationen von verletzten Körperregionen zeigt, dass insbesondere gleichzeitige Verletzungen der oberen und unteren Extremitäten dominieren (32,2 % der Fälle; Abb. 4). Diese Konstellation ist charakteristisch für Explosions- und Splittertraumata, bei denen mehrere Extremitäten gleichzeitig betroffen sind.

Weitere relevante Kombinationen betreffen Kopf/Hals und obere Extremität (7,8 % der Fälle) sowie Becken und untere Extremität (6,7 % Fälle). Ebenfalls jeweils 6,7 % der Fälle entfielen auf Kopf/Hals und untere Extremität. Eine vergleichsweise seltenere Kombination war obere Extremität und Thorax (4,4 % der Fälle).

Die dargestellten Ergebnisse unterstreichen, dass komplexe Mehrfachverletzungen nicht nur mehrere Körperregionen, sondern häufig auch funktionell besonders bedeutsame Bereiche betreffen. Dies verdeutlicht die hohe Relevanz einer interdisziplinären Traumaversorgung, die unfallchirurgische-orthopädische, neurochirurgische, viszeralchirurgische und intensivmedizinische Expertise eng verzahnt.

**Abb. 5:**
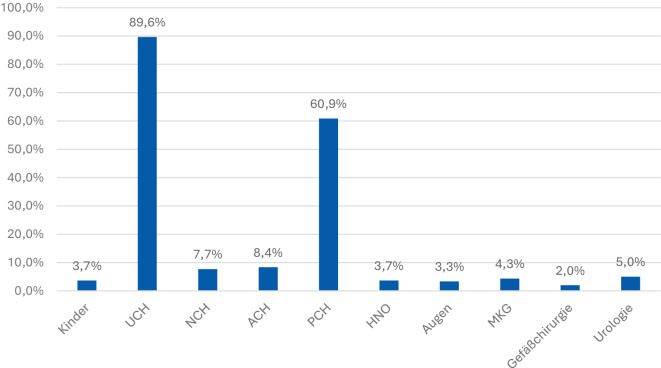
Erforderliche Fachabteilungen für die Behandlung. Überwiegend Unfallchirurgie und plastische Chirurgie, gefolgt von Allgemeinchirurgie und Neurochirurgie

Die Analyse der Fachabteilungen, in denen ukrainische Kriegsverletzte primär versorgt wurden, verdeutlicht die Dominanz chirurgischer Disziplinen (Abb. 5). Mit Abstand am häufigsten war die UCH-Versorgung erforderlich (89,6 % der Fälle), gefolgt von der PCH mit 60,9 % der Fälle. Diese Verteilung spiegelt die hohe Prävalenz komplexer Extremitätenverletzungen sowie rekonstruktiver Eingriffe nach Explosions- und Schussverletzungen wider.

Weitere relevante Fachbereiche sind die NCH, (7,7 % der Fälle) und die viszeralchirurgische Versorgung (ACH, 8,4 % der Fälle), die insbesondere bei Verletzungen von Schädel, Wirbelsäule oder Abdomen eine Rolle spielen.

Seltener waren Behandlungen in anderen Fachrichtungen notwendig: HNO (3,7 % der Fälle), Augenheilkunde (3,3 % der Fälle), MKG-Chirurgie (4,3 % der Fälle), Gefäßchirurgie (2 % der Fälle) und Urologie (5 % der Fälle). Auch die pädiatrische Versorgung war mit 3,7 % der Fälle vertreten, was die – im Vergleich zu Erwachsenen – deutlich geringere Zahl verletzter Kinder widerspiegelt.

Die Ergebnisse unterstreichen, dass die traumatologische und plastisch-rekonstruktive Chirurgie die Hauptlast der medizinischen Versorgung trägt, während spezialisierte chirurgische Fächer punktuell, aber essenziell bei bestimmten Verletzungsmustern beteiligt sind.

**Abb. 6:**
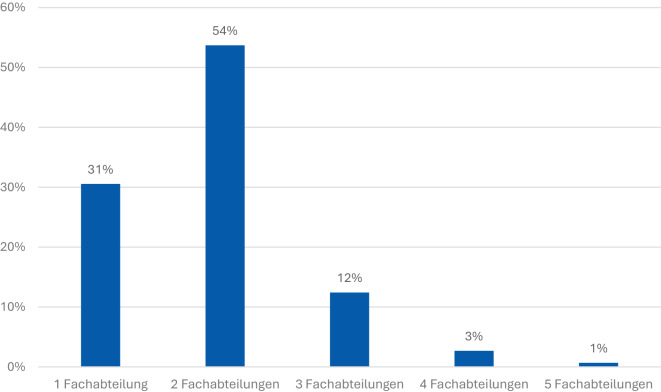
Anzahl beteiligter Fachabteilungen. Mehrheit mit interdisziplinärer Behandlung (zwei Fachabteilungen)

Die Analyse der interdisziplinären Versorgung zeigt, dass nur 31 % der Verletzten durch eine Fachrichtung versorgt werden mussten (Abb. 6). Die Mehrheit der Patienten benötigte eine Vorhaltung mehrerer Fachabteilungen. Am häufigsten waren zwei Fachabteilungen beteiligt (54 % der Fälle). Bei 12 % der Patienten waren drei Fachabteilungen erforderlich, während bei 3 % der Patienten sogar vier Disziplinen involviert waren. Besonders komplexe Fälle mit der Beteiligung von fünf Fachabteilungen traten bei 1 % der Patienten auf.

Diese Verteilung verdeutlicht, dass die Versorgung ukrainischer Kriegsverletzter überwiegend interdisziplinär erfolgt. Der hohe Anteil von Behandlungen mit zwei oder mehr beteiligten Fachrichtungen unterstreicht die Komplexität der Verletzungsmuster – insbesondere im Hinblick auf Extremitätentraumata, kombinierte Verletzungen von Kopf, Thorax und Abdomen sowie die Notwendigkeit rekonstruktiver Maßnahmen. Damit wird die enge Kooperation zwischen Unfallchirurgie, plastischer Chirurgie, Neurochirurgie und weiteren chirurgischen Disziplinen als zentraler Bestandteil der Versorgung deutlich.

**Abb. 7:**
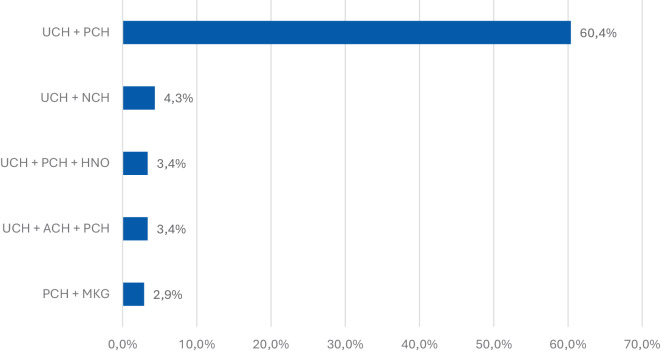
Die 5 häufigsten Kombinationen beteiligter Fachabteilungen. Am häufigsten Unfallchirurgie und plastische Chirurgie gemeinsam

Die Analyse der interdisziplinären Zusammenarbeit zeigt, dass die Kombination aus UCH und PCH mit großem Abstand am häufigsten vorkam (60,4 % der Fälle; Abb. 7). Dies reflektiert die hohe Zahl komplexer Extremitätentraumata, die sowohl unfallchirurgische Stabilisierung als auch plastisch-rekonstruktive Maßnahmen erfordern.

Deutlich seltener traten andere Kombinationen auf: UCH + NCH in 4,3 % der Fälle, UCH + PCH + HNO sowie UCH + Viszeralchirurgie (ACH) + PCH jeweils in 3,4 % der Fälle. Eine vergleichsweise seltene, aber dennoch klinisch bedeutsame Kombination war die Zusammenarbeit von PCH und MKG mit 2,9 % der Fälle.

Diese Ergebnisse verdeutlichen, dass die Versorgung ukrainischer Kriegsverletzter überwiegend durch die enge Verzahnung von unfallchirurgischer und plastisch-rekonstruktiver Expertise geprägt ist. Ergänzend werden bei spezifischen Verletzungsmustern weitere chirurgische Disziplinen hinzugezogen, was die Notwendigkeit einer multidisziplinären Traumaversorgung in spezialisierten Zentren unterstreicht.

Brandverletzungen haben einen relevanten Anteil an den Verletzungen insgesamt (7,7 %). Die Brandverletzten nehmen eine Sonderrolle ein, da sie umfängliche klinische Ressourcen benötigen (Schwerbandverletztenzentren). Hinzu kommt eine lange intensivmedizinische, operative und stationäre Behandlungsdauer. Häufig sind sie mit multiresistenten Keimen besiedelt oder infiziert und sind daher als extrem ressourcenintensiv einzuordnen.

**Abb. 8:**
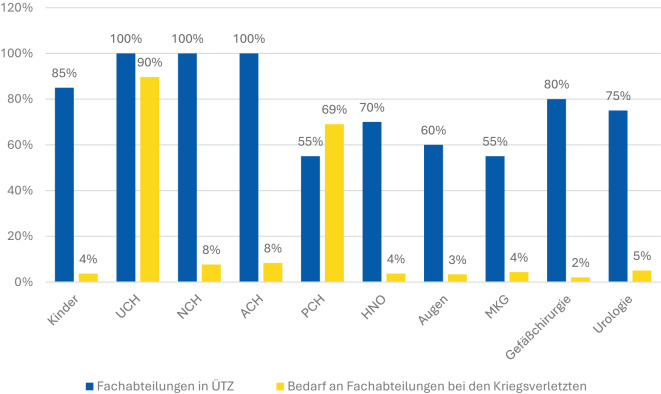
Fachabteilungen in den ÜTZ (TraumaNetzwerk NRW) und deren Inanspruchnahme bei Kriegsverletzungen. Dargestellt ist das Vorhandensein benötigter Fachabteilungen in den überregionalen Traumazentren (ÜTZ) Nordrhein-Westfalen und deren Inanspruchnahme bei ukrainischen Kriegsverletzten

Dargestellt ist das Vorhandensein benötigter Fachabteilungen in den überregionalen Traumazentren (ÜTZ) Nordrhein-Westfalen und deren Inanspruchnahme bei ukrainischen Kriegsverletzten (Abb. 8).

Bei der PCH zeigt sich in den ÜTZ eine Unterdeckung: 55 % der Zentren verfügen über eine entsprechende Einrichtung, obwohl sie bei nahezu 70 % der Kriegsverletzten – inklusive der Brandverletzten – benötigt wurde.

**Abb. 9:**
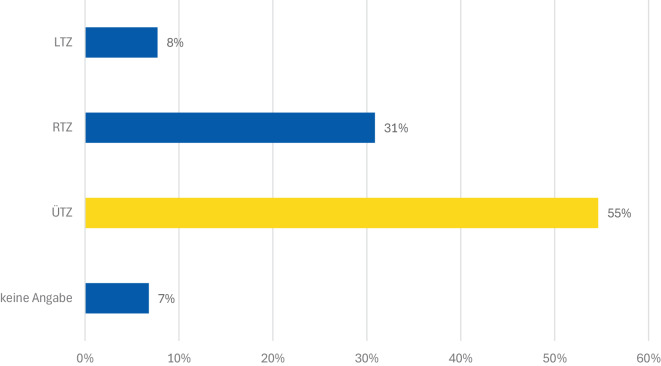
Verteilung der ukrainischen Kriegsverletzten auf die Traumazentren der Traumanetzwerke in NRW

Die tatsächliche Verteilung der in NRW behandelten ukrainischen Kriegsverletzten zeigt eine Konzentration auf die überregionalen Traumazentren (ÜTZ; Abb. 9). Insgesamt wurden 55 % der Patientinnen und Patienten in ÜTZ versorgt, während 31 % in regionalen (RTZ) und 8 % in lokalen Traumazentren (LTZ) behandelt wurden. Diese Verteilung verdeutlicht die zentrale Rolle der überregionalen Traumazentren bei der Versorgung komplexer Kriegsverletzungen. Eine Einschränkung dieser Auswertung besteht darin, dass wir anhand der uns zur Verfügung stehenden Daten nicht eruieren konnten, ob bei einzelnen Verletzten im weiteren Behandlungsverlauf eine Weiterverlegung erforderlich wurde.

## Ausblick und Diskussion

Die Behandlung kriegsverletzter Patientinnen und Patienten stellt das Gesundheitssystem vor besondere Herausforderungen. Diese Patienten gelangen oft erst nach Wochen und Monaten in unsere Behandlung, die meisten Wunden sind mit sog. Problemkeimen (z. B. 4 MRGN) kontaminiert und belasten auch die hiesigen Krankenhäuser.

Eine Limitation dieser Untersuchung ist, dass nur die nach NRW transportierten Patienten ausgewertet werden konnten.

Die Auswertung lässt keine weiteren Schlussfolgerungen zu, da eine entsprechende Vergleichsgruppe aus anderen Regionen fehlen. Somit handelt es sich bei diesem Manuskript lediglich um eine deskriptive Darstellung einer Stichprobe.

Bereits im „Bündnisfall“ wird mit über 1000 behandlungsbedürftigen Patientinnen und Patienten/Tag, die nach Deutschland kommen und hier weiterverteilt werden müssen, gerechnet [2].

Hiervon werden die zivilen Krankenhäuser den Hauptteil der Patienten versorgen müssen. Unklar ist, welche Krankenhäuser diese Menge an Verletzten aufnehmen und versorgen können. Derzeit wird davon ausgegangen, dass dafür wesentlich die Krankenhäuser der Bundeswehr, die Universitätskliniken, die Berufsgenossenschaftlichen Krankenhäuser und weitere Krankenhäuser der Maximalversorgung herangezogen würden.

Die Verteilungsmuster spiegeln lediglich die in diesem Zusammenhang in NRW behandelten Verletzten wider.

Einschränkend muss angemerkt werden, dass die hier ausgewertete Patientenkohorte lediglich die Kriegsverletzten darstellen, die aus der Ukraine in unsere Kliniken gebracht wurden und nicht die gesamte Situation der Verletzungen in der Ukraine darstellen.

In dieser Arbeit wurden im ersten Schritt die Verletzungsverteilung ermittelt, im zweiten Schritt die benötigten Fachabteilungen und im dritten Schritt, welcher Kliniktyp dafür erforderlich ist.

Bei der Auswertung des Patientengutes wurde erkennbar, dass die zentralen chirurgischen Disziplinen (Unfall‑, Neuro- und Viszeralchirurgie) in allen ÜTZ (Krankenhäusern der Maximalversorgung) vertreten sind. Bei der plastischen Chirurgie (PCH) ist jedoch eine deutliche Diskrepanz erkennbar. Nur 55 % der Zentren verfügen über eine entsprechende Abteilung, obwohl sie bei nahezu 70 % der Kriegsverletzten, inklusive der Brandverletzten, benötigt wurde. Diese Lücke unterstreicht den hohen Stellenwert der plastischen und rekonstruktiven Chirurgie in der Versorgung komplexer Kriegsverletzungen und den Bedarf an besserer struktureller Abdeckung in diesem Bereich.

Aufgrund der beobachteten schweren und interdisziplinär zu behandelnden Verletzungsmuster lässt sich erkennen, dass die bisherige chirurgische Ausbildung eine differenzierte Versorgung derartig komplexer Verletzungsmuster bislang noch nicht ausreichend zu gewährleisten scheint.

Aus diesen Gründen sollten die bewährten Fort- und Weiterbildungsformate der medizinischen Fachgesellschaften und der Bundeswehr (DGU, DGAI, DIVI, DGCH etc.) verstärkt genutzt oder sogar neue Weiterbildungsformate geschaffen werden, um auf die Versorgung dieser speziellen Verletzungsmuster ausreichend vorzubereiten.

Zusätzlich müssen die Standardisierung und der Ausbau bewährter telemedizinischer interdisziplinärer Formate (Extremitäten-Boards) intensiviert werden.

Darüber hinaus zeigt sich, dass in Krankenhäusern, die speziell für die komplexeren Verletzungsmuster mit dem Bedarf an mehreren Fachabteilungen ausgerüstet sind, gerade die plastische Chirurgie oft fehlt, obwohl sie sehr häufig benötigt wird.

Zugleich weisen die Daten auf die Relevanz interdisziplinärer Nachsorge hin, die von der Neurochirurgie über die plastische Chirurgie bis hin zur Rehabilitationsmedizin reicht.

Zusätzlich ist die Kompetenz von anderen – nichtoperativen Fachdisziplinen – zwingend erforderlich. Diese beziehen sich wesentlich auf internistische, mikrobiologische, pharmakologische, neurologische, psychiatrische (etc.) Fächer. Die Inanspruchnahme dieser Disziplinen war nicht Gegenstand dieser Untersuchung und wurde nicht erfasst.

Im Ergebnis ist festzuhalten, dass die ukrainischen Kriegsverletzten in den Krankenhäusern in NRW, die in der Lage waren, diese Patienten aufzunehmen, bisher gut versorgt werden konnten. Bei der vorliegenden Auswertung konnten besondere Anforderungen identifiziert werden, die durch Fortbildungsformate und strukturelle Maßnahmen erfüllt werden könnten, sodass die Vorbereitung auf derartige Patienten verbessert werden kann. Insgesamt würde dies zur Etablierung einer robusten und resilienten Versorgungsstruktur und Koordination der Gesundheitsversorgung in Katastrophen- und Verteidigungslagen beitragen.

## Data Availability

Die erhobenen Datensätze können auf begründete Anfrage in anonymisierter Form beim korrespondierenden Autor angefordert werden. Die Daten befinden sich auf einem Datenspeicher am Universitätsklinikum Münster.
